# 3-Amino-5-bromo-2-iodo­pyridine

**DOI:** 10.1107/S1600536808040452

**Published:** 2008-12-06

**Authors:** Kevin D. Bunker, Neal W. Sach, Seiji Nukui, Arnold L. Rheingold, Alex Yanovsky

**Affiliations:** a Pfizer Global Research and Development, La Jolla Laboratories, 10614 Science Center Drive, San Diego, CA 92122, USA; b Department of Chemistry and Biochemistry, University of California San Diego, 9500 Gilman Drive, La Jolla, CA 92093, USA

## Abstract

The reaction of 3-amino-5-bromo­pyridine with *N*-iodo­succinimide in the presence of acetic acid produces the title compound, C_5_H_4_BrIN, with an iodo substituent in position 2 of the pyridine ring. The crystal structure features rather weak inter­molecular N—H⋯N hydrogen bonds linking the mol­ecules into chains along the *z* axis of the crystal.

## Related literature

For structures of *ortho*-iodo­anilines, see: McWilliam *et al.* (2001 ▶); Sandor & Foxman (2000 ▶); Parkin *et al.* (2005 ▶).
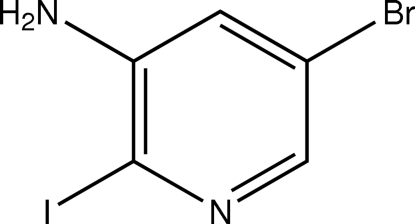


## Experimental

### 

#### Crystal data

C_5_H_4_BrIN_2_
*M* *_r_* = 298.90
Monoclinic, 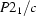

*a* = 4.0983 (12) Å
*b* = 15.172 (4) Å
*c* = 12.038 (3) Å
β = 90.152 (5)°
*V* = 748.5 (3) Å^3^
*Z* = 4
Mo *K*α radiation
μ = 9.53 mm^−1^
*T* = 100 (2) K
0.40 × 0.33 × 0.04 mm

#### Data collection

Bruker APEXII CCD diffractometer
Absorption correction: multi-scan (*SADABS*; Bruker, 2001 ▶) *T*_min_ = 0.234, *T*_max_ = 0.557
3783 measured reflections
1251 independent reflections
1086 reflections with *I* > 2σ(*I*)
*R*_int_ = 0.037

#### Refinement

*R*[*F* ^2^ > 2σ(*F* ^2^)] = 0.032
*wR*(*F* ^2^) = 0.082
*S* = 1.05
1251 reflections
82 parameters
H-atom parameters constrained
Δρ_max_ = 1.33 e Å^−3^
Δρ_min_ = −0.92 e Å^−3^

### 

Data collection: *APEX2* (Bruker, 2007 ▶); cell refinement: *SAINT* (Bruker, 2007 ▶); data reduction: *SAINT*; program(s) used to solve structure: *SHELXS97* (Sheldrick, 2008 ▶); program(s) used to refine structure: *SHELXL97* (Sheldrick, 2008 ▶); molecular graphics: *SHELXTL* (Sheldrick, 2008 ▶); software used to prepare material for publication: *SHELXTL*.

## Supplementary Material

Crystal structure: contains datablocks global, I. DOI: 10.1107/S1600536808040452/rz2275sup1.cif

Structure factors: contains datablocks I. DOI: 10.1107/S1600536808040452/rz2275Isup2.hkl

Additional supplementary materials:  crystallographic information; 3D view; checkCIF report

## Figures and Tables

**Table 1:** Hydrogen-bond geometry (Å, °)

*D*—H⋯*A*	*D*—H	H⋯*A*	*D*⋯*A*	*D*—H⋯*A*
N2—H2*A*⋯N1^i^	0.88	2.16	3.025 (8)	166
N2—H2*B*⋯I1	0.88	2.79	3.259 (5)	115

Symmetry code: (i) 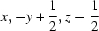.

**Fig. 1.**
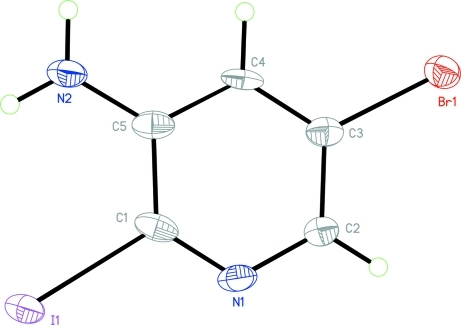
Molecular structure of the title compound, showing 50% probability displacement ellipsoids and atom numbering scheme. H atoms are drawn as circles with arbitrary small radius.

**Fig. 2.**
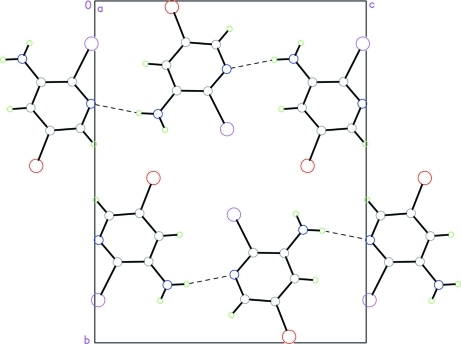
The crystal packing diagram viewed down the x-axis.

## supplementary crystallographic information

### Comment

The reaction of 5-bromo-3-aminopyridine with *N*-iodosuccinimide in the presence of acetic acid leads to iodo-substitution at position 2 of the pyridine ring, as shown by the X-ray study of the title compound (Fig. 1). To the best of our knowledge, this is the first structure of *ortho*-iodoaminopyridine derivative. The N2···I1 distance 3.259 (5) Å is typical for *ortho*-iodoanilines (McWilliam *et al.*, 2001; Sandor & Foxman, 2000; Parkin *et al.*, 2005) and may suggest involvement of the H2B atom in weak intramolecular N2—H2B···I1 interaction (Table 1).

The second `active' H-atom, H2A, participates in the intermolecular H-bond N2—H2A···N1^i^ (symmetry code (i): *x*, 1/2 - *y*, *z* - 1/2; Table 1), which links the molecules into the chains along the *z*-axis of the crystal (Fig. 2). There are no strong halogen···halogen interactions in the structure; the shortest intermolecular I···I distances are 4.091 (1) Å and 4.098 (1) Å.

### Experimental

To a solution of 3-amino-5-bromopyridine (100 mg, 0.56 mmol) in acetic acid (0.1 *M*, 5.61 ml) was added *N*-iodosuccinimide (133 mg, 0.56 mmol) at rt. After 3 h, the reaction was quenched with sat. sodium bicarbonate and extracted 3 times with EtOAc. The organic layers were combined, dried, filtered, and concentrated. The crude residue was subjected to flash chromatography (silica gel, 0–50% EtOAc/heptane). Isolated 93 mg (55%) of 3-amino-5-bromo-2-iodopyridine, as a brown solid. X-ray quality crystals were obtained by slow evaporation of a concentrated chromatography fraction (approx. 30% EtOAc/heptane). ^1^H NMR (400 MHz, DMSO-d_6_) (δ p.p.m.) 5.65 (s, 2 H), 7.16 (d, J = 2.27 Hz, 1 H), 7.67 (d, J = 2.01 Hz, 1 H). ^13^C NMR (101 MHz, DMSO-d_6_) (δ p.p.m.) 106.10, 120.01, 120.92, 137.97, 147.68.

### Refinement

All H atoms were treated as riding with the C—H and N—H distances of 0.95 Å and 0.88 Å respectively; the *U*_iso_(H) were set to 1.2*U*_eq_ of the carrying atom.

### Crystal data

**Table:**

C_5_H_4_BrIN_2_	*F*(000) = 544
*M**_r_* = 298.90	*D*_x_ = 2.652 Mg m^−^^3^
Monoclinic, *P*2_1_/*c*	Mo *K*α radiation, λ = 0.71073 Å
Hall symbol: -P 2ybc	Cell parameters from 2536 reflections
*a* = 4.0983 (12) Å	θ = 2.7–25.3°
*b* = 15.172 (4) Å	µ = 9.53 mm^−^^1^
*c* = 12.038 (3) Å	*T* = 100 K
β = 90.152 (5)°	Plate, colourless
*V* = 748.5 (3) Å^3^	0.40 × 0.33 × 0.04 mm
*Z* = 4	

### Data collection

**Table:**

Bruker APEXII CCD diffractometer	1251 independent reflections
Radiation source: fine-focus sealed tube	1086 reflections with *I* > 2σ(*I*)
graphite	*R*_int_ = 0.037
φ and ω scans	θ_max_ = 25.3°, θ_min_ = 2.2°
Absorption correction: analytical (*SADABS*; Bruker, 2001)	*h* = −4→1
*T*_min_ = 0.234, *T*_max_ = 0.557	*k* = −17→18
3783 measured reflections	*l* = −10→14

### Refinement

**Table:**

Refinement on *F*^2^	Primary atom site location: structure-invariant direct methods
Least-squares matrix: full	Secondary atom site location: difference Fourier map
*R*[*F*^2^ > 2σ(*F*^2^)] = 0.032	Hydrogen site location: inferred from neighbouring sites
*wR*(*F*^2^) = 0.082	H-atom parameters constrained
*S* = 1.05	*w* = 1/[σ^2^(*F*_o_^2^) + (0.037*P*)^2^ + 2.524*P*] where *P* = (*F*_o_^2^ + 2*F*_c_^2^)/3
1251 reflections	(Δ/σ)_max_ = 0.003
82 parameters	Δρ_max_ = 1.33 e Å^−^^3^
0 restraints	Δρ_min_ = −0.92 e Å^−^^3^

### Special details

**Table:**

Geometry. All e.s.d.'s (except the e.s.d. in the dihedral angle between two l.s. planes) are estimated using the full covariance matrix. The cell e.s.d.'s are taken into account individually in the estimation of e.s.d.'s in distances, angles and torsion angles; correlations between e.s.d.'s in cell parameters are only used when they are defined by crystal symmetry. An approximate (isotropic) treatment of cell e.s.d.'s is used for estimating e.s.d.'s involving l.s. planes.
Refinement. Refinement of *F*^2^ against ALL reflections. The weighted *R*-factor *wR* and goodness of fit *S* are based on *F*^2^, conventional *R*-factors *R* are based on *F*, with *F* set to zero for negative *F*^2^. The threshold expression of *F*^2^ > σ(*F*^2^) is used only for calculating *R*-factors(gt) *etc*. and is not relevant to the choice of reflections for refinement. *R*-factors based on *F*^2^ are statistically about twice as large as those based on *F*, and *R*- factors based on ALL data will be even larger.

### Fractional atomic coordinates and isotropic or equivalent isotropic displacement parameters (Å^2^)

**Table:**

	*x*	*y*	*z*	*U*_iso_*/*U*_eq_	
I1	0.18405 (10)	0.37509 (3)	0.48761 (3)	0.02516 (17)	
Br1	−0.56202 (16)	0.01782 (4)	0.28399 (5)	0.0266 (2)	
N2	−0.0278 (15)	0.3306 (4)	0.2326 (4)	0.0298 (13)	
H2A	−0.0808	0.3282	0.1617	0.036*	
H2B	0.0760	0.3769	0.2589	0.036*	
N1	−0.1133 (14)	0.1994 (4)	0.4858 (4)	0.0264 (13)	
C2	−0.2684 (18)	0.1278 (4)	0.4470 (6)	0.0264 (15)	
H2	−0.3243	0.0815	0.4965	0.032*	
C1	−0.0390 (15)	0.2637 (5)	0.4165 (5)	0.0242 (14)	
C5	−0.1071 (15)	0.2622 (5)	0.3019 (5)	0.0227 (14)	
C3	−0.3480 (16)	0.1208 (4)	0.3350 (5)	0.0221 (14)	
C4	−0.2713 (15)	0.1878 (4)	0.2636 (5)	0.0213 (14)	
H4	−0.3303	0.1834	0.1874	0.026*	

### Atomic displacement parameters (Å^2^)

**Table:**

	*U*^11^	*U*^22^	*U*^33^	*U*^12^	*U*^13^	*U*^23^
I1	0.0244 (3)	0.0324 (3)	0.0187 (3)	−0.00094 (16)	−0.00522 (18)	−0.00340 (17)
Br1	0.0285 (4)	0.0274 (4)	0.0238 (4)	−0.0007 (3)	−0.0039 (3)	−0.0027 (3)
N2	0.044 (4)	0.029 (3)	0.016 (3)	−0.002 (3)	−0.008 (3)	−0.001 (2)
N1	0.035 (3)	0.030 (3)	0.015 (3)	0.002 (2)	−0.005 (2)	0.002 (2)
C2	0.035 (4)	0.024 (4)	0.020 (3)	0.000 (3)	−0.007 (3)	0.002 (3)
C1	0.017 (3)	0.036 (4)	0.020 (3)	0.004 (3)	−0.006 (3)	−0.008 (3)
C5	0.017 (3)	0.035 (4)	0.016 (3)	0.006 (3)	−0.001 (2)	−0.002 (3)
C3	0.021 (4)	0.027 (4)	0.018 (3)	0.005 (3)	−0.001 (3)	−0.001 (3)
C4	0.021 (3)	0.033 (4)	0.011 (3)	0.007 (3)	−0.005 (2)	−0.006 (3)

### Geometric parameters (Å, °)

**Table:**

I1—C1	2.102 (7)	C2—C3	1.390 (9)
Br1—C3	1.894 (7)	C2—H2	0.9500
N2—C5	1.371 (9)	C1—C5	1.407 (9)
N2—H2A	0.8800	C5—C4	1.393 (9)
N2—H2B	0.8800	C3—C4	1.368 (9)
N1—C1	1.320 (9)	C4—H4	0.9500
N1—C2	1.342 (9)		
			
C5—N2—H2A	120.0	N2—C5—C4	121.8 (5)
C5—N2—H2B	120.0	N2—C5—C1	122.6 (6)
H2A—N2—H2B	120.0	C4—C5—C1	115.6 (6)
C1—N1—C2	119.3 (5)	C4—C3—C2	119.9 (6)
N1—C2—C3	120.6 (6)	C4—C3—Br1	121.1 (5)
N1—C2—H2	119.7	C2—C3—Br1	119.0 (5)
C3—C2—H2	119.7	C3—C4—C5	120.4 (6)
N1—C1—C5	124.2 (6)	C3—C4—H4	119.8
N1—C1—I1	116.0 (4)	C5—C4—H4	119.8
C5—C1—I1	119.8 (5)		

### Hydrogen-bond geometry (Å, °)

**Table:**

*D*—H···*A*	*D*—H	H···*A*	*D*···*A*	*D*—H···*A*
N2—H2A···N1^i^	0.88	2.16	3.025 (8)	166
N2—H2B···I1	0.88	2.79	3.259 (5)	115

Symmetry codes: (i) *x*, −*y*+1/2, *z*−1/2.

## References

[bb1] Bruker (2001). *SADABS* Bruker AXS Inc., Madison, Wisconsin, USA.

[bb2] Bruker (2007). *APEX2* and *SAINT* Bruker AXS Inc., Madison, Wisconsin, USA.

[bb3] McWilliam, S. A., Skakle, J. M. S., Low, J. N., Wardell, J. L., Garden, S. J., Pinto, A. C., Torres, J. C. & Glidewell, C. (2001). *Acta Cryst.* C**57**, 942–945. 10.1107/s0108270101007053 11498621

[bb4] Parkin, A., Spanswick, C. K., Pulham, C. R. & Wilson, C. C. (2005). *Acta Cryst.* E**61**, o1087–o1089.

[bb5] Sandor, R. B. & Foxman, B. M. (2000). *Tetrahedron*, **56**, 6805–6812.

[bb6] Sheldrick, G. M. (2008). *Acta Cryst.* A**64**, 112–122. 10.1107/S0108767307043930 18156677

